# MicroRNA-19a-3p regulates cell growth through modulation of the PIK3IP1-AKT pathway in hepatocellular carcinoma

**DOI:** 10.7150/jca.37748

**Published:** 2020-02-10

**Authors:** Hai-Xiang Sun, Zhang-Fu Yang, Wei-Guo Tang, Ai-Wu Ke, Wei-ren Liu, Yan Li, Chao Gao, Bo Hu, Pei-Yao Fu, Min-Cheng Yu, Bo-Wen Gao, Ying-hong Shi, Jia Fan, Yang Xu

**Affiliations:** 1 Liver Cancer Institute, Zhongshan Hospital, Fudan University; Key Laboratory of Carcinogenesis and Cancer Invasion (Fudan University), Ministry of Education, Shanghai 200032, China; 2 Department of Liver Surgery and Transplantation, Zhongshan Hospital, Fudan University; Key Laboratory of Carcinogenesis and Cancer Invasion (Fudan University), Ministry of Education, Shanghai 200032, China; 3 Minhang Hospital, Fudan University; Institute of Fudan-Minhang Academic Health System, Minhang Hospital, Fudan University, Shanghai 200032, China; 4 Institute of Biomedical Sciences, Fudan University, Shanghai 200032, China

**Keywords:** microRNA-19a-3p, miR-19a-3p, phosphatidyl inositol 3-kinase interacting protein 1, PIK3IP1, hepatocellular carcinoma, cell growth

## Abstract

There are some controversies about the involvement of microRNA (miR)-19a-3p in hepatocellular carcinoma (HCC) biology, even though many studies have shown that it plays an important role in cancer. In this study, we found that miR-19a-3p is usually overexpressed in HCC tissues compared with corresponding peritumorous tissues, and its expression was associated with tumor size and poor overall survival. MiR-19a-3p promoted cell proliferation significantly, and more cells were found in the S phase. In vivo, miR-19a-3p promoted liver tumor growth, and more HCC cells were found in the active cell cycle. Sequencing and bioinformatics analysis predicted that *PIK3IP1* is a likely target gene of miR-19a-3p, and we next confirmed it by luciferase and rescue assays. Altogether, our data showed an important role of PIK3IP1 downregulation by miR-19a-3p in HCC progression, and the miR-19a-3p-PIK3IP1-AKT pathway may be a potential therapeutic target.

## Introduction

Hepatocellular carcinoma (HCC) is one of the most common cancer types 1 and is the third leading cause of tumor-related deaths in China 2, 3. Even though there have been continuous improvements in screening, diagnosis, and treatment over the past decades, an effective approach to control HCC progression is not available, except for surgical treatment. The 5-year survival rate of patients with liver cancer is only 18% when all stages are considered 1. Therefore, understanding the molecular pathogenesis of HCC and identifying therapeutic targets are extremely urgent goals.

MicroRNAs (miRNAs) are evolutionarily conserved small noncoding RNAs, which are known to play a major role in various biological phenomena, including cell differentiation, immune responses, and cancer 4. Usually, miRNAs regulate gene expression at the post-transcriptional level by blocking mRNA translation or by causing degradation of target mRNAs by binding to their 3′-untranslated regions (3′-UTRs) 5, 6. The miR-17-92 cluster is often upregulated, and individual members of the cluster may have distinct functions 7. miR-19a-3p was identified as a key oncogenic miRNA of the miR-17-92 cluster 8; it is dysregulated in many types of cancer, including gliomas, bladder cancer, breast cancer, and pancreatic, gastric, and laryngeal squamous cell carcinoma, whereas *PPAR-α*, *PTEN*, and *RUNX3* have been identified as its target genes. miR-19a-3p is known to participate in tumor growth, cell survival, and metastasis 9, 10. The involvement of miR-19a-3p in HCC is also well studied, but there are some controversial topics. Some studies have shown that miR-19a-3p is underexpressed in patients with recurrent HCC compared to the nonrecurrence group^11^. It inhibits cell proliferation and promotes more apoptosis in HCC 12.These data indicate its tumor suppressor function but some studies that have been conducted show the opposite effects of miR-19a-3p in HCC. These studies indicate that miR-19a-3p is upregulated in an HCC tumors compared to respective peritumor adjacent tissues 13 and it promotes tumor growth, metastasis, and chemoresistance 14, 15. Therefore, the function of miR-19a-3p in HCC is still unclear and requires more studies.

In the present study, we tested miR-19a-3p expression level in HCC specimens and analyzed the correlation of miR-19a-3p expression levels with outcomes (including recurrence) among 102 patients. We studied the participation of miR-19a-3p in HCC cell growth in a series of in vitro and in vivo experiments. To this end, we screened databases for putative target genes of miR-19a-3p using sequencing and bioinformatics analysis, and investigated the possible molecular mechanism. Our findings confirmed that miR-19a-3p promotes HCC cell proliferation and tumor growth and revealed a new target gene, *PIK3IP1*, thus providing new insights into the molecular functions of miR-19a-3p.

## Materials and Methods

**Patients and HCC samples**. Human liver tissues were obtained from surgically resected specimens of HCC from patients admitted at Zhongshan Hospital, Fudan University. All tumor specimens and matching adjacent nontumorous tissues were snap-frozen in liquid nitrogen after the surgical procedure and stored at -80 ºC. The 102 HCC patients were diagnosed with HCC between 2011 and 2012 and received no chemotherapy or radiotherapy before the resection; the details of these patients are described in Supplementary Table. Clinical data collection and follow-up procedures were performed according to a uniform guideline described previously 16. Overall survival was defined as the time between the dates of surgery and death, and time of recurrence was defined as the interval between the dates of surgery and recurrence. The study protocol was approved by Zhongshan Hospital Ethics Committee, and informed consent was obtained from each patient in accordance with institutional review board protocols.

**Cell culture and transfection.** Huh7 (HCC) cells were purchased from BeNa Culture Collection (Beijing, China). L02, BEL-7402, Hep3B cell lines were obtained from the cell bank of Chinese Academy of Sciences(Shanghai, China), MHCC97H cell lines was previously established in our laboratory. The cells were maintained in Dulbecco's modified Eagle's medium (DMEM) supplemented with 10% of fetal bovine serum. After 12 h of seeding, the cells were placed in a hypoxia workstation (In Vivo 200, Ruskinn Technology, Ltd., Bridgend, UK) for 48 h at 1% O_2._ For a colony formation assay, 1000 cells were seeded in 35 mm dishes and incubated in DMEM with 10% of FBS. Two weeks later, the cells were fixed and stained with 0.1% crystal violet. To generate a lentivirus expressing miR-19a-3p, the precursor sequence was amplified by PCR (the amplicons carried overhangs) and was inserted into the MCS-SV40 expression vector. To knock down the miR-19a-3p expression in Huh7 cells, molecular sponges were used to decrease the miR-19a-3p level. To knock down the expression of PIK3IP1, the small interfering RNA (siRNA) and siRNA control were purchased from Thermo Fisher Scientific. For overexpression of PIK3IP1, the cDNA encoding PIK3IP1 was amplified by RT-PCR and cloned into vector GV358. Huh7 cells were seeded in 6-well plates and incubated overnight, then transfected with the lentivirus according to the manufacturer's instructions.

**RNA extraction, cDNA, RT-PCR,** mRNA sequence **and western blotting**. To analyze miRNA expression, total RNA was extracted with the miRNA Isolation Kit (Life Technologies, Carlsbad, CA), and complementary DNA was synthesized by the miRNA RT Assay (TaqMan). The miR-19a-3p (assay ID 002424, Thermo Fisher Scientific, Inc.) expression level was measured and normalized to small nuclear RNA U6 7. For mRNA analysis, RNA was extracted and purified using the TRIzol Reagent according to the manufacturer's protocol (Thermo Fisher Scientific, Inc.). cDNA was synthesized using random primers, and PCR was carried out with the SYBR Master Mix according to the manufacturer's protocol (Thermo Fisher Scientific, Inc.). *GAPDH* served as a control; the forward primer sequence was 5′-GGGGCTCTCCAGAACATCATCC-3′, and the reverse primer sequence was 5′-ACGCCTGCTTCACCACCTTCTT-3′. Mrna sequence was performed as previously 17 and the data was uploaded to NCBI database (GSE140148). The western blotting protocol was carried out following the method from our previous studies 16. Specific antibodies against the following proteins were employed: cyclin A2 (9869, Cell Signaling Technology, Danvers, MA, USA), Bak (3814s, Cell Signaling Technology), Bax (sc-7480, Santa Cruz Biotechnology, Inc., USA), and PIK3IP1 (sc-365777, Santa Cruz Biotechnology, Inc.).

**In vivo experiment**. Nude mice (4-6 weeks old, male) were kept under specific pathogen-free conditions. Huh7 (HCC) cells (5 × 10^6^) were subcutaneously injected into the flanks of mice. After 4 weeks, the tumor volume reached ~1-2 cm^3^. Next, these tumors were seeded into the liver parenchyma of nude mice under anesthesia after the abdomen was opened up. All the mice were killed 8 weeks later, following which the volume of tumors was calculated.

**Luciferase assay.** PIK3IP1 with a potential miR-19a-3p-binding site or the corresponding mutant site was generated and ligated into luciferase reporter vector GV272. Cells (293T) were seeded in 24-well plates for 24 h incubation. Next, 0.1 μg of a luciferase plasmid harboring the wild-type or mutant miR-19a-3p-binding site in the *PIK3IP1* 3′-UTR and 0.4 μg of the miRNA-encoding plasmid were added into each well together with Lipofectamine 2000 in triplicate. Firefly and *Renilla* luciferase activities were measured consecutively using the Dual-Luciferase Reporter Assay System Kit (Cat. No. E1910, Promega) after 48 h of transfection. *Renilla* luciferase activity was used for normalization.

**Statistical analysis.** Statistical analysis was performed in SPSS 22.0 software for Windows. The chi-squared test was conducted to evaluate differences in categorical variables. Overall survival and time of recurrence were determined by the Kaplan-Meier method. A Cox regression analysis was performed for the multivariate analyses of prognostic factors. Statistical significance was set to p < 0.05.

## Results

### Elevated expression of miR-19a-3p promotes HCC progression and correlates with poor outcomes

**Figure 1:**
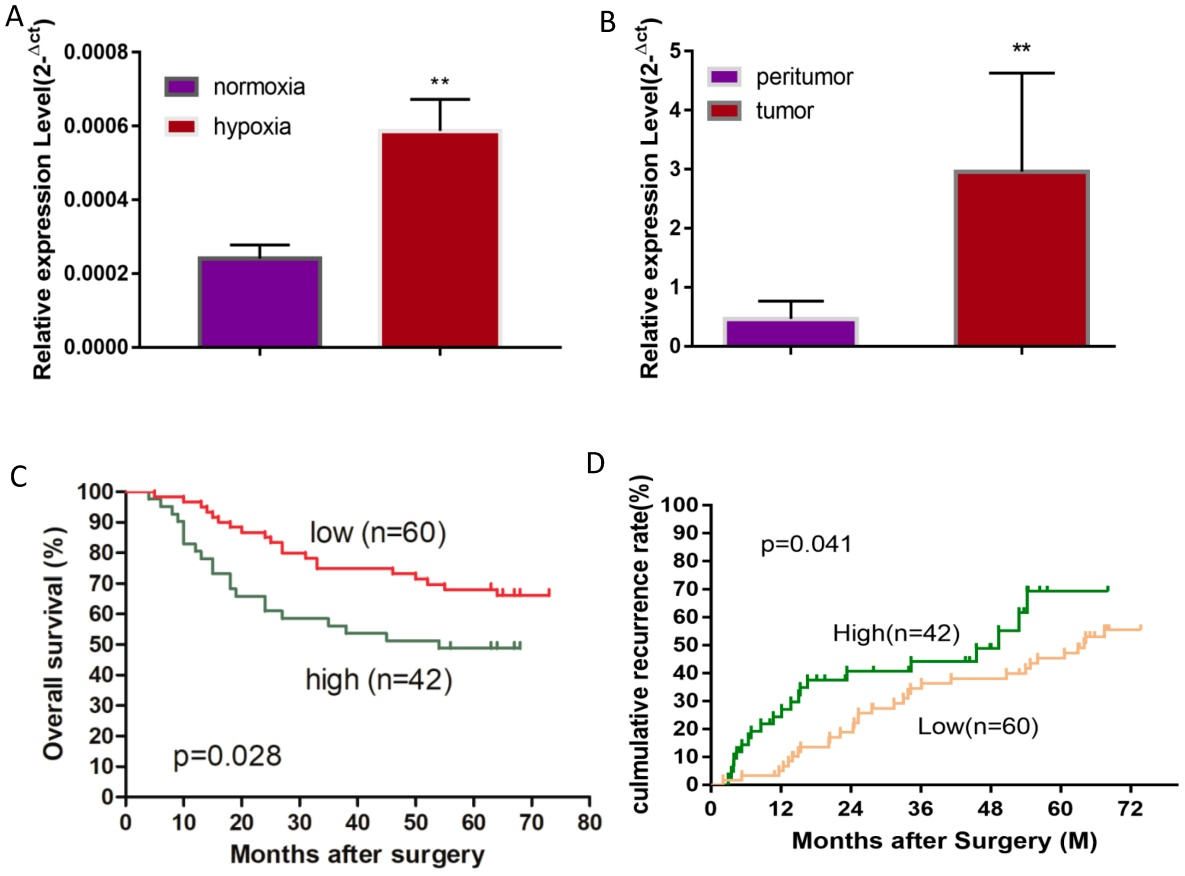
MiR-19a-3p expression in Huh7 cells and HCC tissues. A. Relative miR-19a-3p expression in Huh7 cells cultured for 48 h at 1% O_2_ compared with normoxia (**, p<0.01). B. Relative miR-19a-3p expression in HCC tumors and peritumorous tissues (**, p<0.01). C. The correlation between miR-19a-3p expression levels in HCC tumors and overall survival (p<0.05). D. The correlation between miR-19a-3p expression levels in HCC tumors and the recurrence rate among the patients (p<0.05).

A hypoxic microenvironment is an important characteristic of a tumor and is associated with tumor growth, metastasis, and poor outcomes. Previously, we have identified 58 differentially expressed miRNAs in HCC cell line Huh7 under hypoxia as compared with normoxia, and miRNA-mRNA network analysis revealed that miR-19a-3p is one of the key differentially expressed miRNAs 17. Although miR-19a-3p has been studied in several cancers, the involvement of miR-19a-3p in HCC is still unclear. We initially examined miR-19a-3p expression in Huh7 cells to confirm the miRNA array results, and they showed that ~3-fold upregulation of miR-19a-3p under hypoxic culture conditions occurred as compared to normoxic conditions, which is consistent with the microarray analysis (Figure 1A). It is known that there are many hypoxic areas in a tumor. We next measured the expression levels of miR-19a-3p in HCC tumor samples and their paired peritumor tissue samples by qRT-PCR. Notably, miR-19a-3p turned out to be markedly upregulated in tumor tissue samples compared with the paired peritumor tissue samples (Figure 1B). We then determined the correlation between miR-19a-3p expression and patients' outcomes (including tumor metastasis) among the 102 HCC cases. This analysis indicated that miR-19a-3p expression was positively correlated with tumor size and encapsulation (Supplementary Table 1). Kaplan-Meier analysis indicated that high miR-19a-3p expression was associated with shorter overall survival (Figure 1C) and a higher recurrence rate (Figure 1D) than low miR-19a-3p expression was. More information about patients is provided in supplementary materials (Supplementary Table 2). Collectively, our data suggested that miR-19a-3p might function as an oncogenic miRNA and promote HCC progression.

### MiR-19a-3p promotes HCC growth in vitro and in vivo

**Figure 2:**
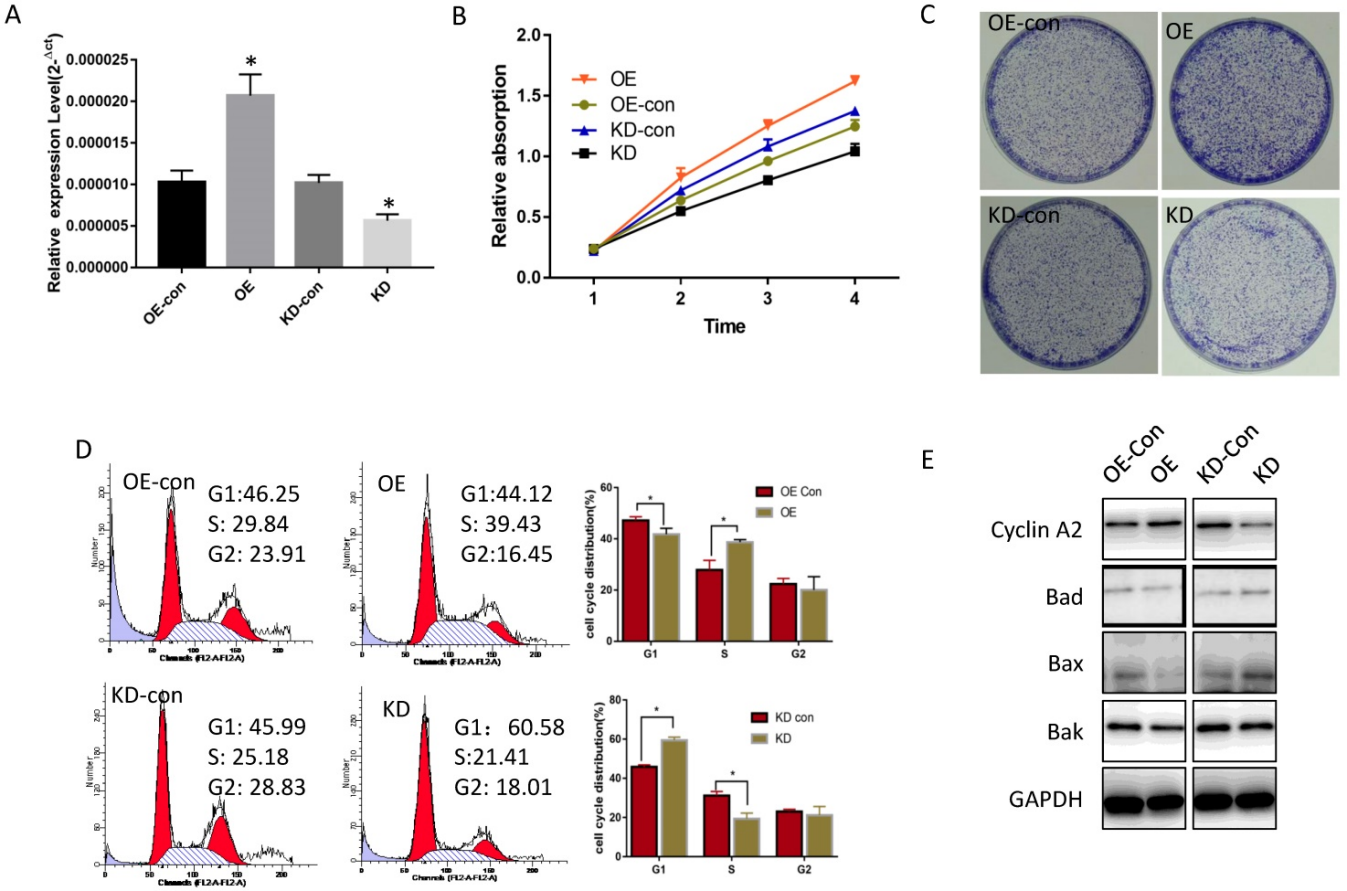
The influence of miR-19a-3p on Huh7 cell growth. A. The effect of miR-19a-3p overexpression and inhibition on miR-19a-3p expression levels (*, p<0.05). B. The action of miR-19a-3p on Huh7 cell growth according to the CCK-8 assay (*, p<0.05). C. The involvement of miR-19a-3p in Huh7 cell growth according to a colony formation assay. D. Flow cytometry. E. Western blot assay.

To determine miR-19a-3p function in HCC, its expression level was tested in several HCC cell lines. Compared with normal hepatic cells, miR-19a-3p was increased in all HCC cell lines (Supplementary Figure 1A). Lenti-miR-19a-3pinhibitor was used to silence miR-19a-3p expression and the lenti-miR-19a-3p-overexpression vector was also constructed. First, miR-19a-3p expression levels were confirmed in the overexpression (OE) group and knockdown (KD) group by qRT-PCR (Figure 2A). The CCK-8 assay results showed that ectopic miR-19a-3p expression greatly enhanced Huh7 growth rates, whereas silencing of miR-19a-3p significantly inhibited HCC cell proliferation (Figure 2B), and a similar result was got in Hep3B cell line(Supplementary Figure 1B). The proliferation-promoting action of miR-19a-3p on Huh7 and hep3B were next confirmed in a colony formation assay (Figure 2C and Supplementary Figure 1C). Cell cycle analysis revealed that high miR-19a-3p expression led to a greater percentage of cells in the S phase, indicating that the G1-S transition was accelerated significantly, whereas miR-19a-3p under-expression yielded the opposite phenotype both in Huh7 and Hep3B cell lines (Figure 2D, Supplementary Figure 1D). Western blot analysis showed that cell cycle-related protein cyclin A2 was upregulated, and apoptosis-related proteins such as Bad, Bax, and Bak were downregulated in the OE group. Contrastingly, the KD group cells had the opposite phenotype (Figure 2E, Supplementary Figure 1E). Altogether, the above results suggested that miR-19a-3p induces HCC cell proliferation in vitro.

**Figure 3:**
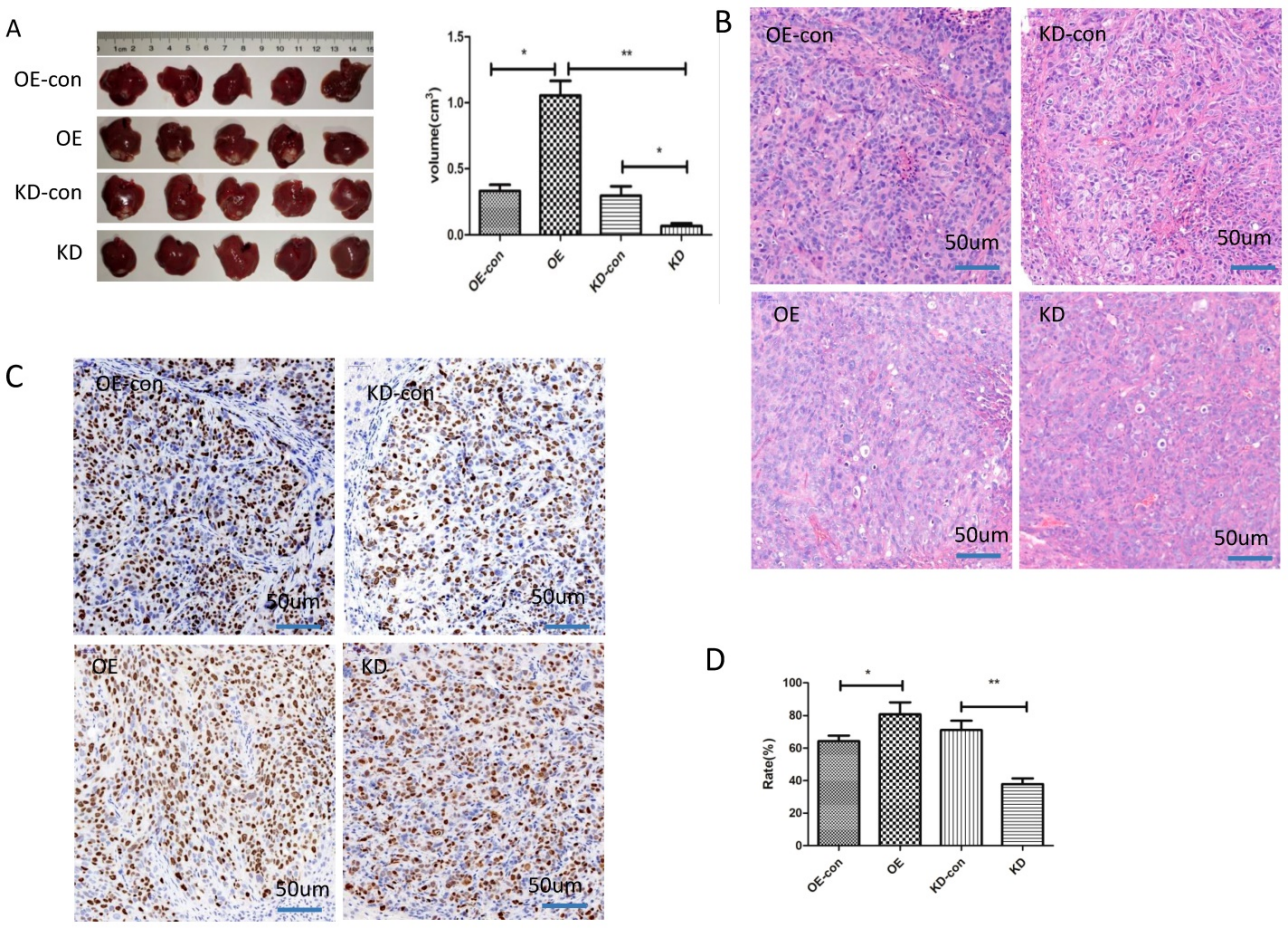
The impact of miR-19a-3p on HCC tumor growth in a xenograft mouse model. A. Tumors with different miR-19a-3p expression levels (*, P<0.05;**, P<0.01). B. H&E staining C&D. The KI67 assay and statistic assay (*, P<0.05;**, P<0.01).

We next studied the influence of miR-19a-3p on HCC growth in vivo. HCC cells with low or high miR-19a-3p expression levels were inoculated into nude mice. The results showed that the tumors in the mice that were injected with miR-19a-3p OE cells were bigger compared with the corresponding control, whereas downregulation of miR-19a-3p expression significantly repressed the tumor growth (Figure 3A). These results are consistent with our in vitro data. Ki67 staining indicated that miR-19a-3p OE yielded more tumor cells that were Ki67-positive (~80%), i.e., more proliferating cells relative to the control were detected. There were only ~37% Ki67-positive tumor cells in the control group. All these data implied that miR-19a-3p significantly promoted HCC tumor growth by improving tumor cell proliferation.

### *PIK3IP1* mRNA is a direct target of miR-19a-3p

**Figure 4:**
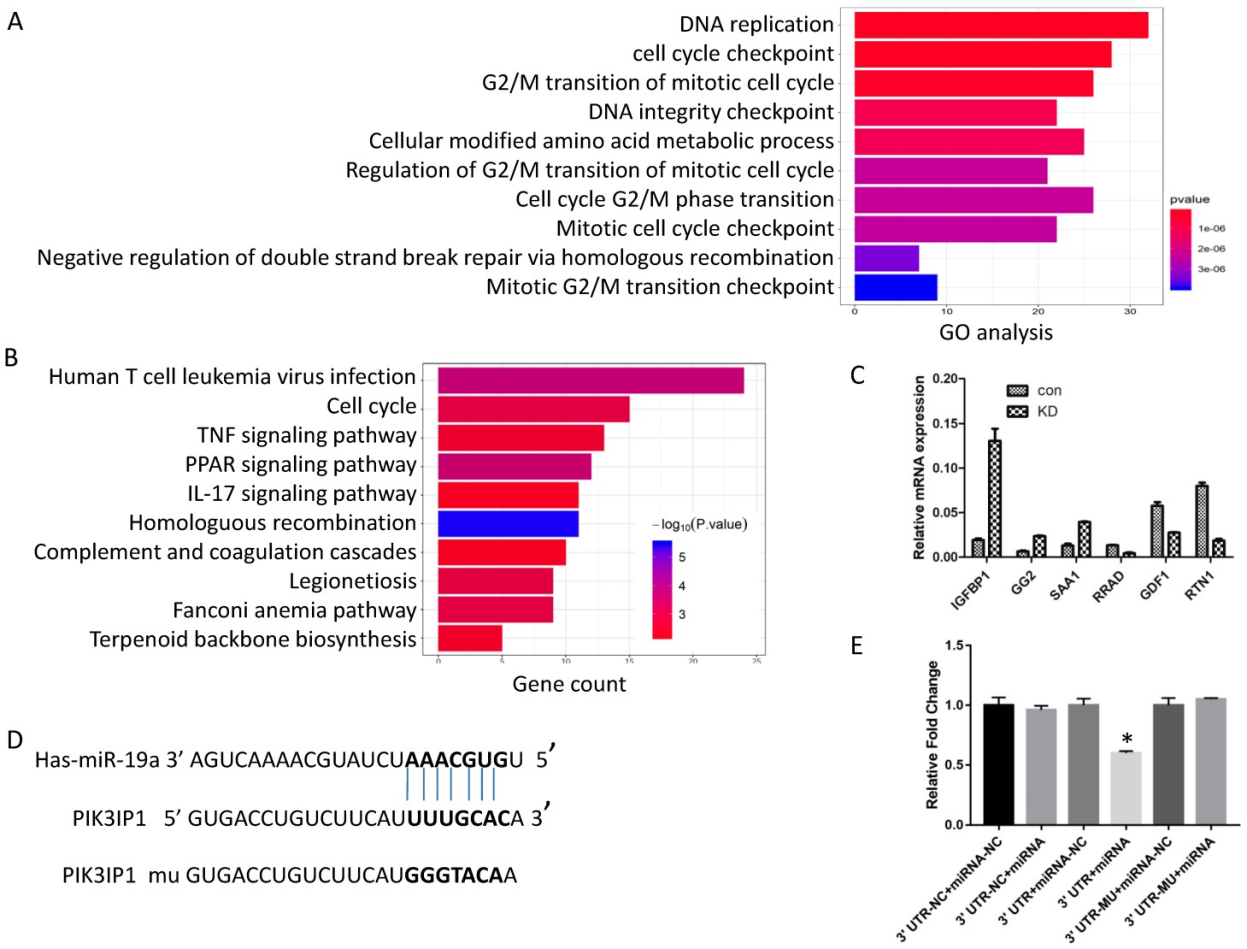
Screening of numerous genes for miR-19a-3p target genes by a sequencing assay, bioinformatics analysis, and a dual luciferase assay. A. GO analysis. B. KEGG pathway analysis. C. RT-PCR verification of the selected genes. D. The putative target sequence for miR-19a-3p in the 3′-UTR of *PIK3IP1*. E. The luciferase assay showed that miR-19a-3p inhibited the luciferase significantly (*,p<0.05).

Generally, miRNAs exert their functions by regulating the expression of target genes. Next, to investigate the possible mechanism of action of miR-19a-3p, we screened the global gene expression changes between miR-19a-3p KD and miR-19a-3p-overexpressing Huh7 cells by high-throughout sequencing the mRNA expression profiles. There were 450 genes that were significantly downregulated, and 431 genes which were upregulated (Supplementary Figure 2A). These genes were next subjected to Gene Ontology (GO) annotation and pathway enrichment analysis. The top 10 GO terms of biological processes were identified, and DNA replication and cell cycle checkpoint were the top two items (Figure 4A). Functional classification of these differentially expressed genes based on the Kyoto Encyclopedia of Genes and Genomes (KEGG) pathway analysis also demonstrated that these differentially expressed genes were highly associated with human T-cell leukemia virus infection, the cell cycle, and TNF signaling pathway, among others (Figure 4B). On the basis of these data, we mainly screened genes involved in cell growth and cell cycle as putative target genes of miR-19a-3p. RT-PCR was carried out to validate the sequencing results (Figure 4C).

In combination with the above sequencing analysis, we searched a number of databases for putative miR-19a-3p target genes, such as microRNA.org, TargetScan, and miRDB, and we found that the 3′-UTR of PIK3IP1 contains a complementary site for the seed region of miR-19a-3p (Figure 4D). To test whether there is a direct interaction between miR-19a-3p and the *PIK3IP1* gene, we performed a dual-luciferase reporter assay using the 3′-UTR of PIK3IP1 and its mutant version (contains a mutation in the miR-19a-3p recognition site). Our data showed that transient transfection of the wild-type PIK3IP1-luc reporter along with miR-19a-3p into Huh7 cells led to a significant decrease of luciferase activity as compared with miR-19a-3p empty vector control, whereas transient transfection of the mutant PIK3IP1-luc reporter together with miR-19a-3p did not yield a luciferase activity change (Figure 4E). All these observations suggested that PIK3IP1 is a direct target of miR-19a-3p in Huh7 cancer cells.

### MiR-19a-3p regulates cell proliferation via the PIK3IP1-PI3K-AKT signaling pathway in HCC

**Figure 5:**
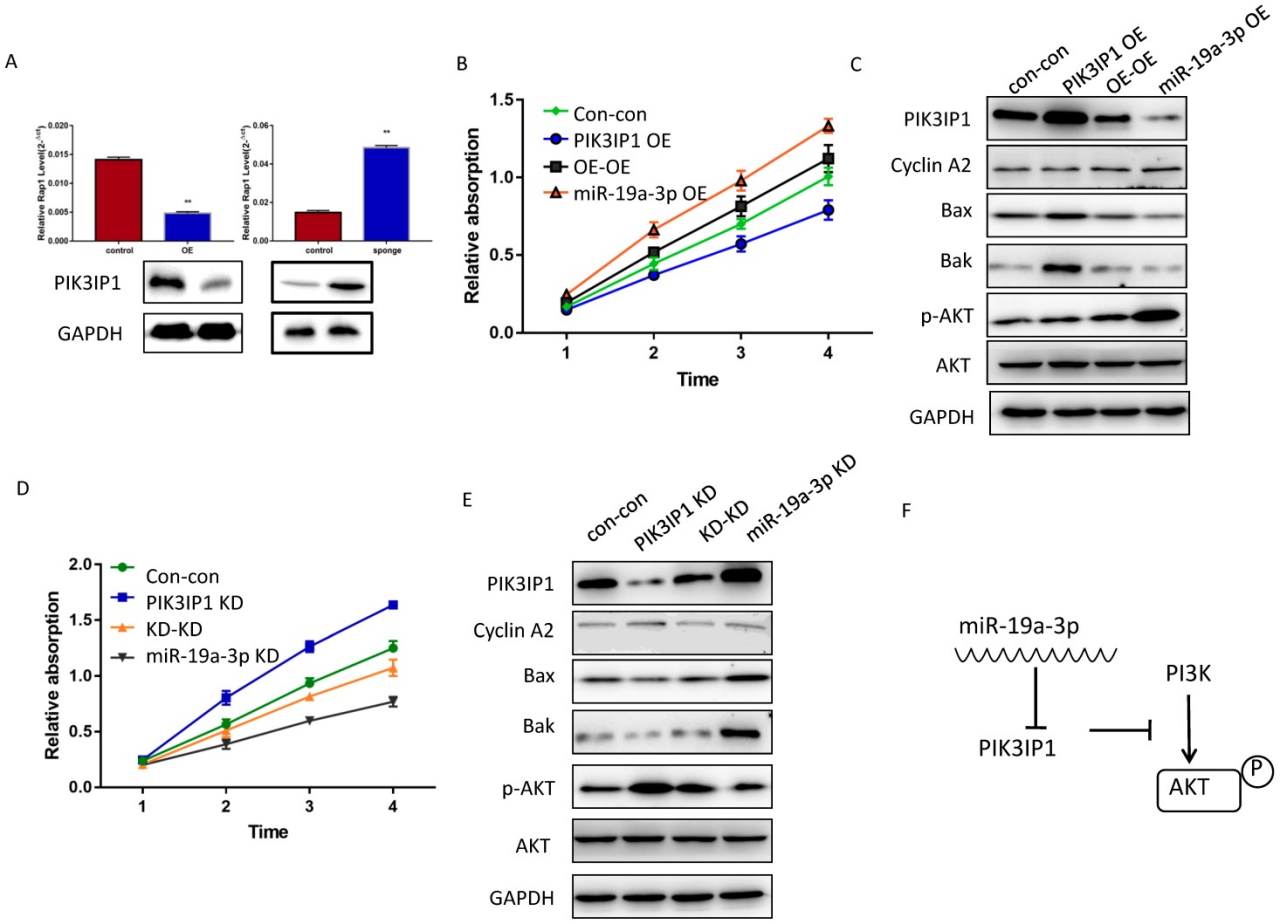
The effect of miR-19a-3p on PIK3IP1. A. PIK3IP1 levels in different cells ( **, P<0.01). B. The CCK-8 assay for determining the role of PIK3IP1 expression in miR-19a-3p OE cells. C A western blot assay of expression of relevant proteins in PIK3IP1 OE cells and controls. D. The CCK-8 assay for determining the role of PIK3IP1 expression in miR-19a-3p KD cells. E. The western blot assay of expression of relevant proteins in PIK3IP1 KD cells and controls. F. Schematic model of cross talk between miR-19a-3p, PI3K and p-AKT.

To determine whether miR-19a-3p regulates PIK3IP1 expression in Huh7 cancer cells, we evaluated the PIK3IP1 expression level in miR-19a-3p OE and miR-19a-3p KD groups. The level of PIK3IP1 was markedly reduced by ectopic expression of miR-19a-3p, whereas PIK3IP1 expression was significantly increased by the KD of miR-19a-3p (Figure 5A). PIK3IP1 staining of the mouse tumors also showed that elevated miR-19a-3p expression led to low expression of PIK3IP1, whereas the KD of miR-19a-3p yielded the opposite phenotype (Supplementary Figure 3A).

PIK3IP1 is a transmembrane protein that possesses an intracellular domain homologous to the p85 regulatory subunit of PI3K; PIK3IP1 downregulates PI3K activity by binding to a PI3K subunit through a specific domain 18. PIK3IP1 is underexpressed in HCC tumors compared with the corresponding peritumor tissues (Supplementary Figure 3B), consistent with previous studies, which show that PIK3IP1 suppresses hepatocyte proliferation and HCC progression 19. To test whether the dependence of cell proliferation on miR-19a-3p was determined by PIK3IP1 downregulation, rescue experiments were performed. Huh7 cells with miR-19a-3p OE were transfected with lenti-PIK3IP1 or the empty vector. CCK-8 assays revealed that the significant proliferation induced by miR-19a-3p was partially reversed by introduction of PIK3IP1 (Figure 5B), and cell cycle-related protein cyclin A2 was downregulated apoptosis-related protein expression levels increased (Figure 5C). Conversely, the KD of PIK3IP1 mimicked the effects of miR-19a-3p and significantly increased cellular proliferation (Figure 5D); relevant proteins manifested the same pattern of expression (Figure 5E). AKT and phospho- AKT were also quantified to determine the possible mechanism of action of the miR-19a-3p-PIK3IP1 axis. We found that cells with a high miR-19a-3p level featured low PIK3IP1 expression and an increased p-AKT amount. When we restored the PIK3IP1 expression level in these cells, the p-AKT amount decreased significantly (Figure 5C). This result was confirmed by knocking down miR-19a-3p and PIK3IP1 in Huh7 cells (Figure 5E). All these results indicated that miR-19a-3p promotes cell proliferation by downregulating PIK3IP1 and upregulating p-AKT expression levels (Figure 5F).

## Discussion

MiRNAs are members of the noncoding RNA family and have been reported to fine-tune the expression of thousands of mRNAs simultaneously through either mRNA degradation or repression of translation. Some oncogenic genes such as MYC 20 and some tumor suppressor genes such as p53 21 have been proven as miRNA target genes. Meanwhile, miRNA may work as a downstream effector of some important transcription factors such as STAT3 22 and of some long noncoding RNAs (lncRNA). Homeostatic imbalance of lncRNAs, miRNAs, and cancer-regulatory factors results in biochemical and physiological alterations inside the cell, and these are critical parameters for cancer progression 23.

miR-19a is a member of the miR-17-92 miRcluster and the dysregulation of the miR-19a is previously found to be associated with multiple types of cancer, including breast cancer^24^, prostate cancer^25^, and breast cancer^10^. Studies showed that miR-19a inhibitor increased p53, Bax and caspase3 expression and decreased the Bcl-2 expression. miR-19a directly suppressed PPARalpha, promoted cell proliferation, tumorigenesis and inflammation by FOXO1 in glioma 3, 9. PTEN is shown as one of target genes, it induced cell apoptosis and negatively regulate PI3K/AKT pathway 8. miR-19a may also played important oncogenic role in HCC. miR-19a-3p was shown to be upregulated in HCC specimens compared with matched tumor free tissues, it promoted metastasis and chemoresistiance of HCC via repressing PTEN^15^. miR-19a overexpression promoted cellular proliferation in hep3B and hepG2 cell lines that were neutralized by addition of panobinoustat 26. We confirmed miR-19a-3p oncogenic role by depressed PIK3IP1 expression, a negative regulator of PI3K. However, there are one study showed miR-19a was downregulated in HCC by analysis biological data acquisition and integration from literature retrieval and inhibit cyclin D1 expression 12. Different type of HCC cell lines were used in these studies, and maybe was the reason for the discrepancies of those results. The heterogeneity of HCC tissue and character of patients may also were reasons, as it is known that miRNAs exert specific actions in a context of microenvironment specific dependent manner. Additionally, tissue collected, frozen in a short time and deposit time were also important because miRNA is easily degraded.

PI3K is a well-known regulator of cell division, motility, and survival in most types of cells, and intact PI3K signal transduction is required for proper liver function and development. Aberrant PI3K signal transduction is implicated in liver tumorigenesis, and PI3K pathway constituents are usually altered in human liver cancer. Studies suggest that liver cancer features the highest percentage of cases with a PIK3CA mutation 27, and another component of the PI3K pathway, PTEN, is downregulated in HCC 28. Some studies have shown that *PTEN* is a direct target gene of miR-19a-3p. Here, we found that *PIK3IP1* mRNA is a direct target of miR-19a-3p, and high expression of miR-19a-3p caused downregulation of PIK3IP1 in HCC tissues compared with the adjacent normal tissues. Those studies indicate that miR-19a-3p may be one of the critical elements of the PI3K pathway in liver cancer cells.

MiRNA genes are widespread in all the genomes, and miRNAs may function as a prognostic factor. Compared with other cell types, hepatocytes have been demonstrated to easily take up oligonucleotides, with the implication that miRNA inhibition or replacement might be effective as a potential therapeutic approach. Our results support the notion that inhibition of miR-19a-3p may be a promising approach for sustaining sufficient expression of PIK3IP1 and may be a therapeutic strategy against HCC growth.

## Supplementary Material

Supplementary figures.
