# Supplementary material for: MicroRNA-19a-3p regulates cell growth through modulation of the PIK3IP1-AKT pathway in hepatocellular carcinoma
Source: J Cancer. 2020 Feb 10;11(9):2476–84. doi: 10.7150/jca.37748 (PMC7066004; doi:10.7150/jca.37748)
Supplement: Supplementary file 1 — Supplementary figures. [file jcav11p2476s1.pdf]

Supplementary Figure 1. A. miR-19a-3p expression level in different HCC cell lines. B. CCK8 assay of miR-19a-3p role in hep3B. C. Colony formation assay of miR-19a-3p role in hep3B. D. cell cycle analysis. E. Western blot assay.

Supplementary Figure 2. Expression of various genes in miR-19a-3p KD cells and control cells.

Supplementary Figure 3. A. PIK3IP1 expression in xenograft mouse model with different miR-19a-3p expression levels. B. PIK3IP1 expression in human HCC tumor samples and peritumorous normal tissue samples.

Table 1. Correlation Between the Factors and Clinicopathologic Characteristics in HCC (n=102)

Clinicopathological indexes		Mir 19a		
		**Low**	**High**	**P**
**Age(year)**	**≤50**	19	12	0.828
	**>50**	41	30	
**Sex**	**Female**	5	9	0.08
	**Male**	55	33	
**HBeAg**	**Negative**	48	38	0.178
	**Positive**	12	4	
**HBV-DNA**	**Negative**	43	20	0.022
	**Positive**	17	22	
**AFP (ng/ml)**	**≤20**	38	20	0.155
	**>20**	22	22	
**TB(umol/L)**	**≤20**	55	40	0.697
	**>20**	5	2	
**ALT(U/L)**	**≤50**	49	33	0.801
	**>50**	11	9	
**PALB(g/L)**	**≤0.25**	49	33	0.801
	**>0.25**	11	9	
**ALB(g/L)**	**≤35**	8	1	0.078
	**>35**	52	41	
**GGT (U/L)**	**≤60**	30	25	0.421
	**>60**	30	7	
**PT(s)**	**≤13**	48	5	0.419
	**>13**	12	41	
**Liver cirrhosis**	**No**	39	23	0.311
	**Yes**	21	19	
**Tumor size(cm)**	**≤5**	38	18	0.046
	**>5**	22	24	
**Tumor number**	**Single**	37	25	0.840
	**Multiple**	23	17	
**Microvascular invasion**	**Absence**	44	25	0.197
	**Present**	16	17	
**Tumor encapsulation**	**Complete**	43	21	0.037
	**None**	17	21	
**Tumor differentiation**	**I+II**	33	25	0.689
	**III+IV**	27	17	
**TNM stage**	**I**	35	19	0.229
	**II+III**	25	23	

AFP, alpha-fetoprotein; GGT, gamma glutamyl transferase; TNM, tumor-node-metastasis;ALT, alanine transaminase; TB, total bilirubin; PALB,prealbumin;ALB, albumin; AKP, alkaline phosphatase; *Fisher's exact tests; chi-square tests for all other analyses.

Table 2. Univariate and Multivariate Analyses of Prognostic Factors in HCC (n =102)

	TTR		OS	
**Variable**	**HR (95% CI)**	**P**	**HR (95% CI)**	**P**
**Univariate analysis**				
Age, year (≤50 versus >50)	0.611(0.341-1.094)	0.098	1.332(0.653-2.720)	0.431
Sex (female versus male)	1.196(0.533-2.683)	0.665	1.533(0.743-3.163)	0.248
HBeAg (negative versus positive)	1.360(0.655-2.824)	0.409	0.984(0.461-2.100)	0.966
HBV-DNA(negative versus positive)	1.445(0.801-2.609)	0.221	1.994(1.080-3.684)	0.027
AFP, ng/ml (≤20 versus >20)	1.841(1.057-3.206)	0.031	1.387(0.752-2.560)	0.295
TB(umol/L) (≤20 versus >20)	1.107(0.396-3.089)	0.847	0.742(0.179-3.078)	0.681
ALB(g/L)(≤35 versus >35)	7.267(0.9984-52.900)	0.051	2.192(0.529-9.081)	0.279
ALT(U/L)(≤50 versus >50)	1.262(0.627-2.542)	0.514	1.556((0.780-3.105)	0.210
PALB(g/L) (≤0.25 versus >0.25)	0.601(0.268-1.344)	0.215	0.546(0.214-1.392)	0.205
AKP(U/L)(≤125 versus >125)	1.341(0.530-3.394)	0.536	1.558(0.611-3.530)	0.353
PT(s) (≤13 versus >13)	1.047(0.530-2.067)	0.895	0.919(0.386-2.186)	0.848
Liver cirrhosis (no versus yes)	1.472(0.818-2.649)	0.198	2.132(1.152-3.945)	0.016
Tumor size, cm (≤5 versus >5)	2.120(1.216-3.697)	0.008	2.388(1.250-4.560)	0.008
Tumor number (single versus multiple)	1.969(1.140-3.401)	0.015	2.581(1.375-4.843)	0.003
Microvascular invasion (no versus yes)	2.473(1.409-4.339)	0.002	2.016(1.086-3.471)	0.026
Tumor encapsulation (complete versus none)	0.478(0.276-0.827)	0.008	0.479(0.259-0.885)	0.019
Tumor differentiation (I + II versus III + IV)	1.203(0.676-2.139)	0.529	1.386(0.750-2.563)	0.297
TNM stage (I versus II III)	1.939(1.106-3.399)	0.021	2.408(1.246-4.656)	0.009
Mir 19a (low versus high)	1.815(1.016-3.241)	0.033	1.924(1.574-3.553)	0.028
**Multivariate analysis**				
HBV-DNA(negative versus positive)	NA		1.453(0.768-2.749)	0.251
AFP, ng/ml (≤20 versus >20)	1.475(0.830-2.623)	0.186	NA	
Liver cirrhosis (no versus yes)	NA		1.752(0.738-4.157)	0.204
Tumor size, cm (≤5 versus >5)	1.839(0.961-3.522)	0.066	1.584(0.763-3.292)	0.217
Tumor number (single versus multiple)	1.913(0.961-3.522)	0.063	1.940(0.836-4.503)	0.123
Microvascular invasion (no versus yes)	3.280(1.423-7.562)	0.005	1.565(0.674-3.636)	0.297
TNM stage (I versus II III)	0.578(0.212-1.572)	0.283	1.167(0.436-3.125)	0.758
Mir 19a (low versus high)	1.662(0.874-3.160)	0.121	1.964(1.018-3.793)	0.044
Tumor encapsulation (complete versus none)	0.475(0.269-0.841)	0.011	0.517(0.249-1.071)	0.076

Cox proportional hazards regression model. AFP, alpha-fetoprotein; GGT, gamma glutamyl transferase; TNM, tumor-node-metastasis;ALT, alanine transaminase; TB, total bilirubin; PALB, prealbumin; ALB, albumin; AKP, alkaline phosphatase; HR, hazard ratio; CI, confidential interval; NA, not adopted.

Supplementary Figure 1

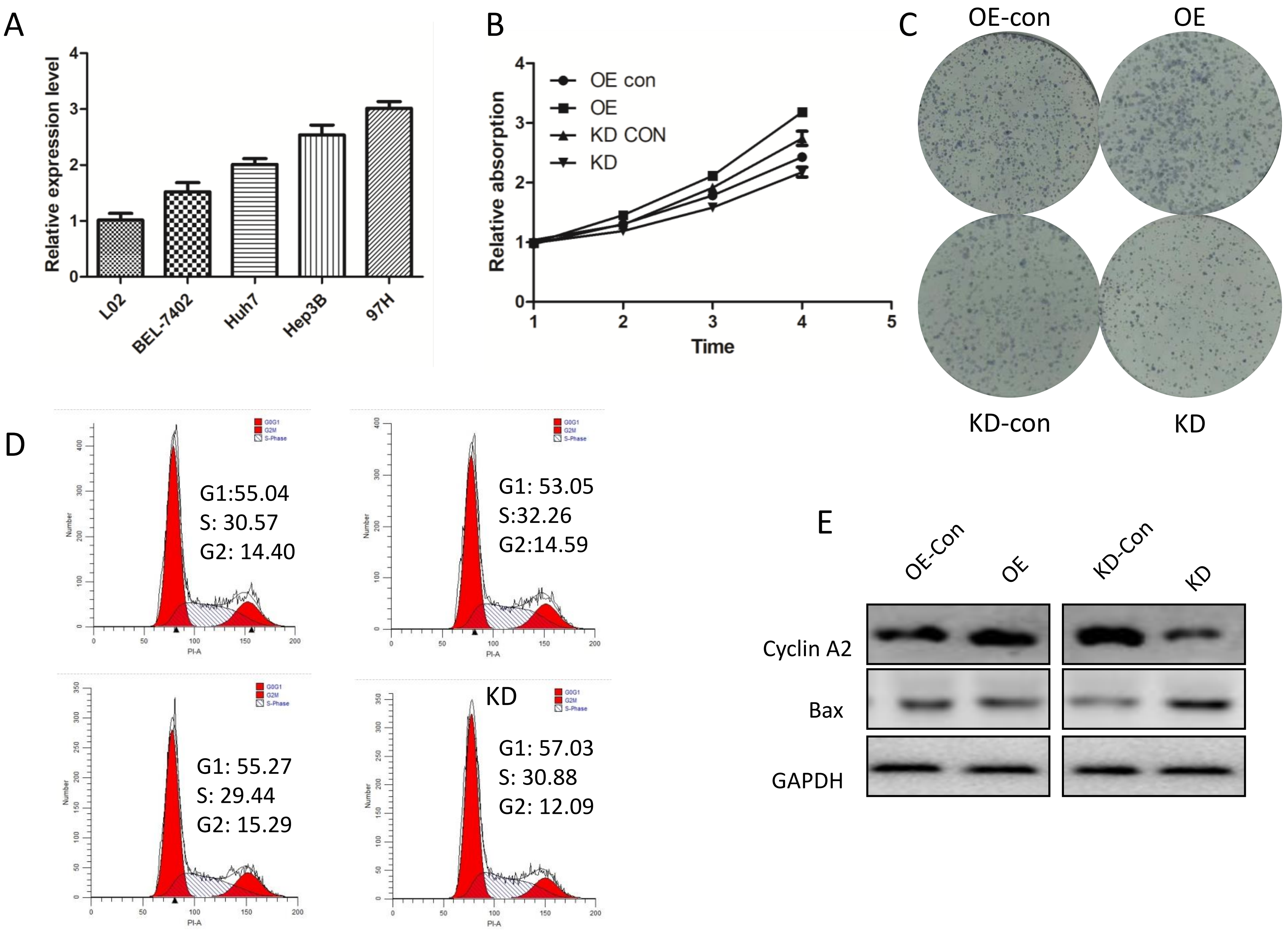

Supplementary Figure 2

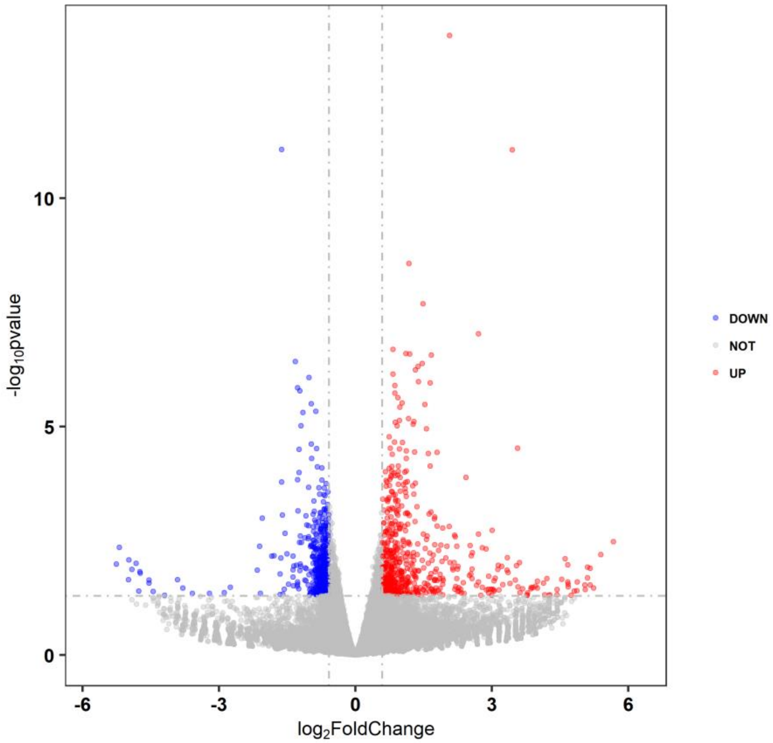

Expression of various genes in miR-19a-3p KD cells and control cells.

Supplementary Figure 3

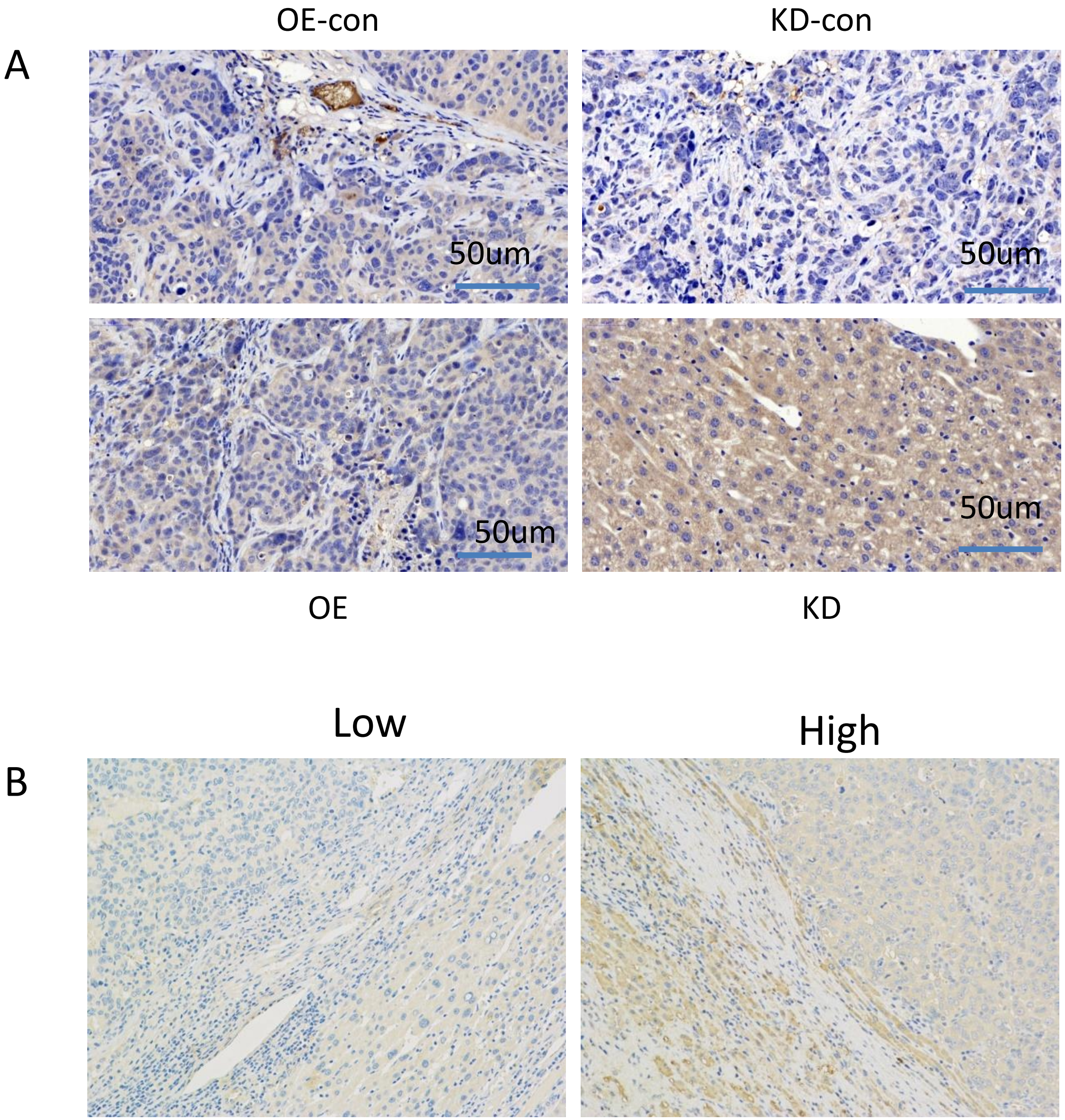

PIK3IP1 expression in HCC tissue. A. xenograft mouse model B. Human HCC
